# Application of therapeutical nanoparticles with neutrophil membrane camouflaging for inflammatory plaques targeting against atherosclerosis

**DOI:** 10.1016/j.mtbio.2024.101397

**Published:** 2024-12-10

**Authors:** Ningnannan Zhang, Tianzhu Zhang, Jintang Feng, Jian Shang, Beibei Zhang, Qingyang Dong, Zhang Zhang, Chunyang Sun

**Affiliations:** a Department of Radiology and Tianjin Key Laboratory of Functional Imaging, Tianjin Medical University General Hospital, Tianjin, 300052, PR China; b Department of Environmental Medicine, Tianjin Institute of Environmental and Operational Medicine, Tianjin, 300050, PR China; c Department of Magnetic Resonance Imaging, The First Affiliated Hospital of Zhengzhou University, Zhengzhou, 450002, PR China

**Keywords:** Biomimetic nanoparticles, Neutrophil, Atherosclerosis, Theranostics, Targeted pharmacotherapy

## Abstract

Atherosclerosis is the leading cause of cardiovascular disease and myocardial infarction. Precise and effective plaque targeting is a major objective for therapeutic outcomes throughout various stages of atherosclerosis. Inspired by the natural recruitment of neutrophils in atherosclerotic plaques, we fabricated a simvastatin (ST)-loaded and neutrophil membrane-cloaked nanoplatform (NNP^ST^) for enhancing localized payload delivery and atherosclerosis management. The resulting NNP^ST^ mimicked neutrophil function and significantly decreased macrophage‐mediated phagocytosis to prolong its own circulation time in the blood. Compared to pristine nanoparticles (NP^ST^) without a membrane coating, NNP^ST^ achieved better plaque targeting in ApoE^−/−^ mice, as indicated by neutrophils actively recruited in atherosclerotic lesions. The higher plaque homing with NNP^ST^ was monitored by dynamic fluorescence/magnetic resonance (MR) dual-modality imaging. The results further showed that NNP^ST^ efficiently prevented atherosclerosis development mainly by suppressing local inflammatory macrophages, and the percentage of plaques in the entire aortic area was reduced to 4.75 ± 1.48 % following NNP^ST^ treatment. A biosafety assessment indicated that the biomimetic NNP^ST^ induced no noticeable toxicity in the body. This approach of neutrophil membrane-camouflaged nanoparticles offers new opportunities to various therapeutic agents for on-demand delivery in neutrophil-involved inflammatory diseases.

## 1 Introduction

Atherosclerosis (AS) is underpinned by lipid accumulation and inflammation in the arterial walls [[1], [2], [3]]. With AS progression, inflammatory reactions promote both subintimal collagen exposure and low-density lipoprotein deposition, suddenly resulting in lethal disorders without perceptible signs, such as acute aortic syndrome or ischemic stroke [[4], [5], [6], [7]]. Statins, which principally reduce blood lipids by inhibiting 3-hydroxy-3-methylglutaryl coenzyme A (HMG-CoA) reductase activity, are the first-line treatment in the clinic for primary and secondary AS prevention [8,9]. Although existing evidence has suggested that statins exhibit anti-inflammatory and plaque-stabilizing functions, oral administration of statins are nonspecifically retained in plaques, which accounts for their poor therapeutic outcomes in targeted lesions [10,11]. In addition, the severe side effects of statins *in vivo* are unavoidable, mainly because of the long-term treatment [12]. Hence, precise and specific statin delivery to inflammatory plaques *via* versatile strategies is of great significance for effective AS therapy.

Fortunately, advances in nanotechnology suggest a passive targeting strategy for AS treatment *via* extravasation through leaky vasculature and the subsequent inflammatory cell-mediated sequestration (ELVIS) effect, similar to the EPR effect in solid tumors [[13], [14], [15]]. Despite great promise, the plaque targeting of most current nanocarriers is deficient because of both the clearance by the immune system and random accumulation manner during blood circulation [16,17]. In recent years, biomimetic technology regarding cell membrane camouflaging has enabled the synthetic nanoparticles (NPs) with distinctive functions, such as prolonged blood circulation and specific lesion targeting, that are inherit to the original cells [18,19]. Pioneer erythrocyte membrane-camouflaged NPs have been proposed to achieve the protective clearance from the reticuloendothelial system (RES) [20,21]. By fusing brain metastatic B16 cell membranes, Liu et al. enabled the NP crossing of the blood–brain barrier in heterologous glioma while reducing toxicity to normal organs *in vivo* [22]. Moreover, cell membrane-derived biomimetic NPs have garnered increasing interest in detecting and treating AS. Red blood cell, platelet or macrophage membrane-mimicking NPs have been engineered to manage AS successfully [[23], [24], [25], [26]].

Neutrophils, the most abundant leukocytes in peripheral circulation, are the first defenders against inflammatory signaling in the innate immune system [[27], [28], [29]]. Their purpose is to respond quickly to microbial infections and to trap and kill invading pathogens. Conclusive evidence has demonstrated that neutrophils appear in atherosclerotic plaques and causally contribute to AS progression [30]. Neutrophil adhesion to chemokine-decorated endothelial cells is activated and promoted upon transient contact and interaction [31]. In the inflammatory atherosclerotic plaques, free-flowing neutrophils bind to selectins (e.g., E-selectin, P-selectin) on the surface of endothelial cells and then roll on the endothelium, governed by various chemokine receptors and cell adhesion molecules [32]. After capture, neutrophils actively crawl across endothelial cells *via* cell–cell connections, ultimately migrating into inflammatory lesions through the transluminal route [33]. Liu et al. utilized neutrophil membrane-coated ZIF-8 nanoparticles to achieve precise delivery of anti-miR-155 for the treatment of atherosclerotic inflammation [34]. Li et al. developed a novel PtdSer-modified neutrophil membrane-based liposome (PtdSer-NM-Lipo/Fer-1) for targeted delivery of Fer-1 to atherosclerotic lesions, effectively inhibiting AS progression and ferroptosis [35]. Compared to statins, the safety of these drugs and nanocarriers still needs further confirmation. As statins and PLGA are FDA-approved, exploring biomimetic nanocarriers camouflaged with sophisticated cell membranes to specifically deliver statin drugs is still highly desired for AS treatment. Additionally, current research lacks non-invasive monitoring of plaques, leading to poor feedback in the assessment of clinical treatment outcomes.

**Scheme 1:**
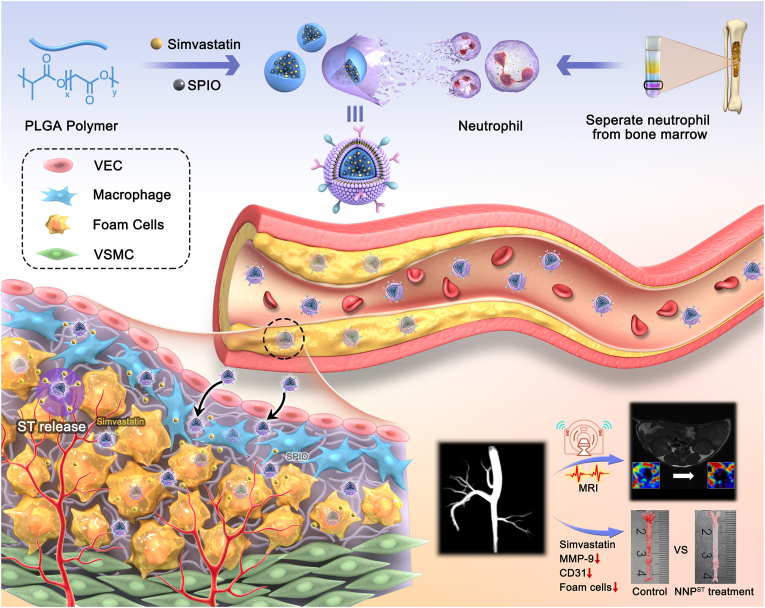
Schematic illustration of NNP^ST^ fabrication and its theranostic effect on AS development. Neutrophil membranes separated from bone marrow were cloaked on the surface of inner PLGA cores as the outer shell. After intravenous administration, the membrane proteins guided the NNP^ST^ to accumulate in inflammatory atherosclerotic plaques, and MRI showed a notable plaque signal reduction at 24 h post-injection. Based on the anti-inflammatory effect of ST, NNP^ST^ remarkably inhibited plaque area and AS progression.

Inspired by the inflammatory tissue recruitment of neutrophils, we designed and constructed biomimetic nanoparticles with a neutrophil membrane camouflaging (NNP) for AS theranostics (Scheme 1). The inner core of the NNP was assembled with the poly(lactic-co-glycolic acid) (PLGA) homopolymer to encapsulate simvastatin (ST) and superparamagnetic iron oxide (SPIO), which are anti-inflammatory and MRI agents, respectively. Membranes were isolated from the bone marrow neutrophils of mature mice and used as the outer biomimetic shell. Owing to the camouflage conferred by this innate immune cell membrane, the NNP get rid of the shackles of the immune system and markedly delayed ST clearance from the blood circulatory system. Clearly, key proteins retained on the neutrophil membranes effectively guided the NNPs to inflammatory lesions. Active NNP enrichment in atherosclerotic plaques was visualized by 9.4 T MR/fluorescence bimodal imaging. Finally, the effectiveness of the NNP^ST^ was systematically examined both *in vitro* and *in vivo*, which clearly showed that NNP^ST^ minimized nonspecific toxicity in normal organs and produced advanced inhibitory effects on AS development.

## 2 Method

### 2.1 Materials

Simvastatin, Fe(acac)_3,_ benzyl ether, oleylamine, and PLGA (MW:66000–107000, 75:25) were purchased from Aladdin (Shanghai, China). Percoll® was purchased from GE Healthcare. 3,3′-dioctadecyloxacarbocyanine perchlorate (DiO), 1,1′-dioctadecyl-3,3,3′,3′-tetramethylindocarbocyanine perchlorate (DiI), 4′,6-diamidino-2-phenylindole (DAPI), lipopolysaccharides (LPS), Membrane Protein Extraction kits and BCA protein assay kit were obtained from Beyotime Institute of Biotechnology. Cell counting kit-8 (CCK-8) were provided by Shanghai Saint-Bio Co. Ltd. Antibody special for mouse PSGL-1 (anti-rabbit) was obtained from Absin Bioscience, and TLR4 (anti-rabbit) special for mouse was purchased from Cell Signaling Technology, Inc. The antibodies special for mouse CD31, CD68, matrix metalloproteinase-9 (MMP-9), α-SMA, and goat horseradish peroxidase (HRP)-*anti*-rabbit were purchased from Abcam.

RAW 264.7 (macrophage) cells were provided by the Institute of Biochemistry and Cell Biology of the Chinese Academy of Sciences (Shanghai, China), and cultivated in DMEM medium containing antibiotics (penicillin 100 U/mL and streptomycin 100 μg/mL) and 10 % fetal bovine serum (FBS).

Male ICR mice (8 weeks), ApoE^−/−^ mice (6 weeks) and male C57BL/6 mice (8 weeks) were obtained from Beijing HFK Biotechnology Co., Ltd. All animal experiments were conducted in accordance with the guidelines approved by the Institutional Animal Care and Use Committee at Tianjin Medical University General Hospital (IRB2021-DWFL-143).

### 2.2 Isolation of neutrophils and membranes

Mouse neutrophils were collected from the bone marrow of ICR mice using a modified gradient centrifugation method [36]. Briefly, bone marrows were flushed from bones of hind legs using RPMI 1640 medium. The suspension was then centrifuged (2000 rpm, 5 min, 4 °C) and resuspended in phosphate-buffered saline (PBS, pH 7.4). Erythrocytes in the sample were lysed with red blood cell lysis buffer and placed over 55 %, 65 % and 78 % of Percoll® solution. Samples were centrifuged at 500 g for 0.5 h and the mature neutrophils from the interface of the 65 % and 78 % gradient layers were collected. The purity of neutrophils was determined using both conjugated CD11b antibody (Abcam) and Ly6g antibody (Abcam). Then the neutrophil membrane was collected by using Membrane Protein Extraction Kit. Finally, the pellet was quantified using a BCA kit and stored at −80 °C for use.

### 2.3 Preparation of ST, DiI and SPIO-loaded PLGA nanoparticles

PLGA nanoparticles containing ST were prepared by a single emulsion method. Briefly, ST (4 mg) and PLGA (20 mg) were co-dissolved in CHCl_3_, the mixture was dropped into 20 mL of 1.5 % (w/v) PVA solution and sonicated at 100 W for 5 min (sonication for 5 s, intervals for 5 s) in an ice bath. Following the rotary evaporation, the suspension was centrifuged at 4000 × rpm for 5 min to remove the free PLGA and ST. Finally, the nanoparticles were separated by centrifugation at 14,500 rpm for 25 min and washed three times with ddH_2_O, the obtained nanoparticles were denoted by NP^ST^. The oleylamine-modified SPIO nanocrystals were synthesized as the previous report (Fig. S1) [37]. To prepare the fluorescently labeled or SPIO nanocrystals loaded nanoparticles, DiI or SPIO were encapsulated based on the similar route, and the obtained nanoparticles were denoted by NP^DiI^ or NP^SPIO^, respectively.

### 2.4 Preparation of neutrophil membrane coating nanoparticles

To obtain neutrophil membrane vesicles (NMV), the neutrophil membrane materials (1 mg/mL) were physically extruded through 400 nm (10 passes) and 200 nm (10 passes) polycarbonate membranes in sequence, respectively. The resulting NMV were mixed with NP^ST^, NP^DiI^ or NP^SPIO^ at a 1:1 of core-to-membrane protein weight ratio and sonicated for 5 min in a bath sonicator. The mixture was then extruded through a 200 nm polycarbonate membrane for ten passes to obtain NNP^ST^, NNP^DiI^ or NNP^SPIO^.

### 2.5 Nanoparticles uptake by macrophages

RAW 264.7 cells were seeded at a density of 2 × 10^5^ cells per well and cultivated with DMEM medium containing 10 % FBS for 24 h at 37 °C. 100 μg of NP^DiI^ or NNP^DiI^ were added and co-incubated with RAW 264.7 cells for 0.5, 1, 2 and 4 h, respectively. Afterwards, the cells were washed with PBS, collected, fixed with 4 % paraformaldehyde, and analyzed by BD Accuri flow cytometry. Meanwhile, to visualize the cellular uptake, RAW 264.7 cells were washed, fixed, and stained by DAPI before CLSM observation.

### 2.6 Inflammation-targeting ability *in vitro*

To determine the inflammatory targeting ability of biomimetic NNP, RAW 264.7 cells were seeded in 12-well plates at a density of 2 × 10^5^ cells per well and incubated with 50 μg/mL LPS for 24 h prior the incubation with NP^DiI^ or NNP^DiI^. Following the cultivation for 4 and 6 h, the cells were washed for three times and subjected to flow cytometry to measure the mean fluorescence intensity. In addition, the cells incubated with biomimetic nanoparticles were fixed, stained by DAPI, and observed by CLSM.

### 2.7 *In vivo* targeting to atherosclerotic plaque

For *in vivo* fluorescence imaging, male ApoE^−/−^ mice (6 weeks old) were fed with a high-fat diet for 3 months. Then they were injected intravenously with DiI-labeled formulations (i.e., NP^DiI^ or NNP^DiI^). After 12 and 24 h post-injections, the mice were sacrificed, and both complete aorta and major organs were isolated. Subsequently, DiI fluorescence in atherosclerotic plaque regions from the collected aorta and the major organs was measured by IVIS imaging system (PerkinElmer, USA). Finally, cross-sections of the aortic roots were then stained with primary anti-mouse CD68 antibody for CLSM observation.

### 2.8 Magnetic resonance imaging of atherosclerotic plaque *in vivo*

ApoE^−/−^ mice were fed a high-fat diet for six months to induce AS and then randomly divided into two groups (n = 3). MR images were acquired using a 9.4 T scanner equipped with a Rat head surface coil (Biospin, Bruker, Germany). Mice were first anaesthetized with 1.5 % isoflurane and received *i.v.* injection with NNP^SPIO^ or NP^SPIO^ ([Fe] = 3 mg/kg), and then T_2__TurboRARE and T_2_map_MSME sequences were acquired after 6, 12 and 24 h post-injection. T_2_map_MSME sequence captures 12 echo images at different effective TE (TE_eff_) times to generate T_2_ map images for quantifying the T_2_ values in tissues, so the enrichment of magnetic nanoparticles in plaques can accurately quantified. Sequences parameters are as follows: T_2__TurboRARE: echo time (TE) = 23 ms, repetition time (TR) = 1835 ms, field of view (FOV) = 35 × 35 mm, matrix = 320 × 320, number of averages (NA) = 6, slice thickness = 0.4 mm, respiratory-gated: ON; T_2_map_MSME: TE_eff_ = 7.5, 15, 22.5, 30, 37.5, 45, 52.5, 60, 67.5, 75, 82.5, and 90 ms, TR = 3068 ms, FOV = 35 × 35 mm, matrix = 192 × 192, NA = 1, slice thickness = 0.4 mm, respiratory-gated: ON. Manually outline the region of interest (ROI) corresponding to the AS plaque on T_2_ map images and measure the mean T_2_ value.

### 2.9 Atherosclerosis treatment *in vivo*

Six-week-old ApoE^−/−^ mice were randomly divided into 4 groups that were treated with different formulations and dosed for 1 month by tail vein injection every three days after 12 weeks of a cholesterol-rich diet feeding. Mice were intravenously injected with saline, free ST, NP^ST^ or NNP^ST^ ([ST] = 1 mg/kg), respectively. The body weight was monitored throughout the treatment period. After the treatment, all mice were euthanized and the whole aortas were collected and fixed with 4 % paraformaldehyde. Afterwards, the extent of atherosclerotic plaques in each group was visualized by Oil red O (ORO) staining. Furthermore, the frozen section of atherosclerotic plaque in the aortic root was determined by ORO staining. The atherosclerotic plaques in the aortic and aortic root were also individually analyzed by using Image J software.

### 2.10 Histology and immunohistochemistry analysis of plaque

At the end of the treatment, aortic roots from mice with various treatments were collected and fixed with 4 % paraformaldehyde. Then paraffin-embedded sections were prepared with 10 μm of the cross-section interval. The necrotic core was determined by toluidine blue staining. MASSON staining method was used to determine collagen content in aortic arch root plaque. For immunohistochemistry analysis, sections of aortic root were stained with CD31, CD68, MMP-9, CD68 or α-smooth muscle actin (α-SMA) antibodies for determining the neovessels, macrophages, MMP-9 or smooth muscle cells (SMCs), respectively. The quantitative histological and immunohistochemical analyses were performed using Image J software.

### 2.11 *In vivo* safety evaluation

After the above-mentioned treatment, blood was collected using EDTAK_2_ spraying tubes, and blood parameters were analyzed immediately by automatic hematology analyzer (BC-2800vet, Mindray, UK). The serum biochemistry analyses on liver, kidney and plasma lipids were performed using an automated analyzer platform (BS-430, Mindray, UK). Additionally, the histological sections of the heart, kidney, liver, spleen and lung of ApoE^−/−^ mice were stained with hematoxylin and eosin (H&E).

## 3 Results and discussion

### 3.1 Preparation and characterization of ST and SPIO-loaded biomimetic nanoparticles

**Fig. 1:**
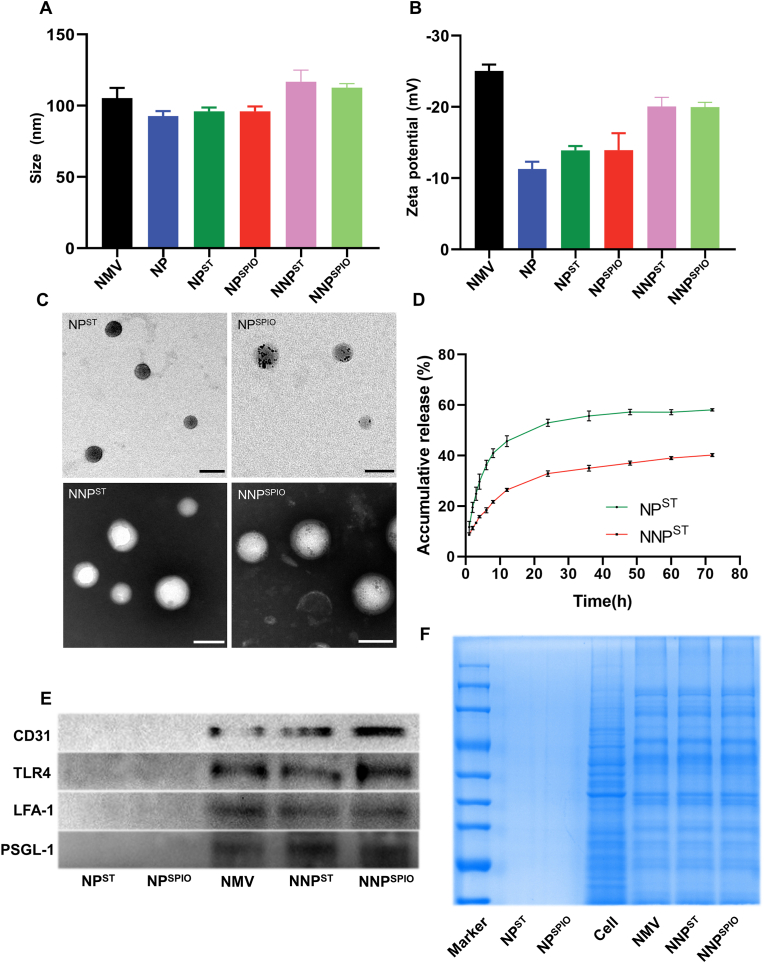
The diameter (A) and zeta potentials (B) of NMV, NP, NP^ST^ and NMV- camouflaged nanoparticles. (C) TEM images of NP^ST^, NP^SPIO^, NNP^ST^ and NNP^SPIO^. The scale bar is 100 nm. (D) *In vitro* ST release of NP^ST^ and NNP^ST^ at pH 7.4 (n = 3). Western blotting (E) analysis of NMV, NP^ST^, NP^SPIO^, NNP^ST^ and NNP^SPIO^. (F) SDS-PAGE.

To construct a neutrophil-mimicking theranostic platform, we first prepared a ST-loaded inner PLGA core (NP^ST^) using the single emulsion method. Further, neutrophils were separated from mouse bone marrow and purified *via* density gradient centrifugation using Percoll® (>90 % purity, Fig. S2). The neutrophil membranes were isolated using a membrane protein extraction kit. Then, the neutrophil membranes were coated on the NP^ST^ surface to obtain biomimetic NNP^ST^ through a cascade extrusion method using 200- and 400-nm filter membranes. While conducting plaque imaging *in vivo*, the loading of SPIO nanocrystals into a polymeric core ensured the integration of MR imaging function for NNP^SPIO^. The average sizes and zeta potentials of NMV and various nanoparticles were determined using dynamic light scattering (DLS, Fig. 1A and B). Compared to NP^ST^ (96.1 nm and −13.9 mV), the increased hydrodynamic diameter and reduced zeta potential of NNP^ST^ (116.8 nm and −19.9 mV) indicated successful coating of neutrophil membranes on the NP^ST^ surface. The DLS results of SPIO-loaded nanoparticles were comparable to those of NNP^ST^. As shown in Fig. 1C, TEM observation confirmed that NNP^ST^ and NNP^SPIO^ displayed a core–shell morphology. Further, the thickness of the monolayer surface shell was ∼9 nm, which agrees with that of the neutrophil membranes. As an MRI agent, SPIO exhibiting a diameter of ∼10 nm was found to be distributed in both NP^SPIO^ and NNP^SPIO^.

**Fig. 2:**
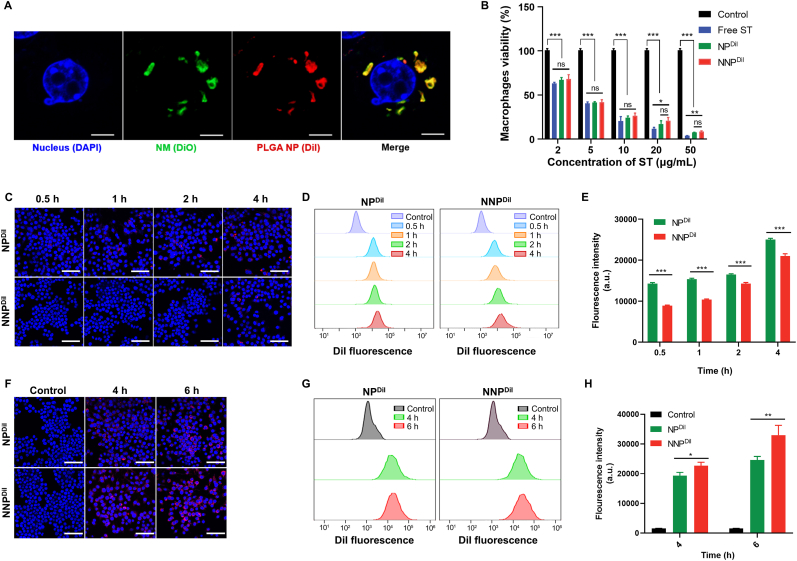
(A) The cellular uptake of NNP by RAW 264.7 cells. The outer NM was labeled by DiO and inner PLGA core was labeled by DiI, respectively. The scale bar is 10 μm. (B) The anti-proliferation activities of ST, NP^ST^ and NNP^ST^ against RAW 264.7 macrophages (n = 3). ∗*p* < 0.05, ∗∗*p* < 0.01, ∗∗∗*p* < 0.001 and *ns*, no significance. (C) CLSM images of the RAW 264.7 cells treated with NP^DiI^ and NNP^DiI^ for 0.5, 1, 2 and 4 h. The scale bar is 50 μm. (D) Semi-quantitative analysis of NP^DiI^ and NNP^DiI^ uptake by flow cytometry. (E) The quantitative cellular uptake of NP^DiI^ and NNP^DiI^ in RAW 264.7 cells (n = 3), ∗∗∗*p* < 0.001. CLSM observation (F) and the mean fluorescence intensity (G) of LPS-stimulated RAW 264.7 macrophages incubated with NP^DiI^ and NNP^DiI^ for 4 and 6 h. (H) The intracellular DiI fluorescence in LPS-stimulated RAW 264.7 macrophages incubated with NP^DiI^ and NNP^DiI^ for 4 and 6 h.

To further verify the successful coating of neutrophil membranes, we encapsulated DiI into the PLGA core and labeled the neutrophil membranes using DiO. The merged fluorescence images of NNP revealed an extensive signal indicating the fusion of green neutrophil membranes and a red polymeric core (Fig. S3). Meanwhile, we incubated RAW 264.7 cells with dual-fluorophore-labeled NNP for 4 h. According to the CLSM visualization, both fluorescence signals exhibited accurate colocalization (primarily distributed in phagosomes and lysosomes) (Fig. 2A), demonstrating that the neutrophil membranes of NNP remained stable even after cellular internalization.

The membrane proteins are essential for neutrophil function. Further, we analyzed the protein spectrum of NMV and various nanoparticles using Coomassie Blue staining to examine whether both NNP^ST^ and NNP^SPIO^ could simulate the biological activity of neutrophils (Fig. 1F). Compared to NP^ST^ and NP^SPIO^, where membrane coating was absent, most of the proteins on the neutrophil membrane were successfully transferred and retained in NNP^ST^ or NNP^SPIO^. Notably, there was no significant difference in proteins among NMV and neutrophil-mimicking nanoparticles. Moreover, western blotting proved the existence of LFA-1, PSGL-1, TLR4, and CD31 proteins in both NNP^ST^ and NNP^SPIO^ (Fig. 1E). Among these proteins, the interaction of LFA-1 with ICAM-1 expressed in vascular endothelium regulates neutrophil rolling, arrest, and transmigration on inflamed endothelium [38]. PSGL-1 exhibits a special affinity with selectin P and is the primary ligand of selectin L and E. Its interactions with selectin molecules promote cell adhesion during inflammatory responses [39,40]. Other proteins (i.e., TLR4 and CD31) are crucial in the pathogenesis of AS [41,42]. These results show that representative proteins on neutrophil membranes are retained during extrusion, endowing the biological functions of both NMV-decorated nanoparticles was potentially comparable to those of neutrophils.

Poor water solubility of ST (0.03 mg/L) greatly hinders its clinical application [43]; however, loading ST into polymeric nanoparticles could overcome this obstacle. The ST loading content of NNP^ST^ was found to be 5.04 % using HPLC analyses, suggesting NNP^ST^ considerably improved ST solubilization. As a delivery system, we studied the ST release rate in NNP^ST^ (Fig. 1D). At pH 7.4, both NP^ST^ and NNP^ST^ exhibited a sustained ST release pattern, and the cumulative ST release of NP^ST^ and NNP^ST^ reached 58.07 % ± 0.43 % and 40.23 % ± 0.51 %, respectively. The slower ST release of NNP^ST^ could be attributed to outer neutrophil-membrane coating, which is in line with the previous reports.

### 3.2 Anti-atherosclerotic effects in RAW 264.7 macrophages

The interaction between nanoparticles and immune cells is essential in blood clearance and biodistribution of intended lesions *in vivo* [44]. Recent studies have indicated that nanoparticles with neutrophil-membrane camouflaging can inhibit the phagocytosis of macrophages [19]. Therefore, DiI was used to label different nanoparticles, and the uptake efficiency of NP^DiI^ and NNP^DiI^ by RAW 264.7 macrophages during different time periods *in vitro* was studied using semiquantitative flow cytometry. As shown in Fig. 2D and E, macrophages internalized less NNP^DiI^ at each time point than NP^DiI^, suggesting that neutrophil-membrane coating assisted the NNP to evade macrophage clearance. Subsequent 10.13039/501100007874CLSM observation supported this viewpoint because only trace red fluorescence could be found after NNP^DiI^ incubation (Fig. 2C).

Inflammation is a common physiological and pathological change during AS progression, and it plays prominent roles at all stages of AS [2]. We further explored whether NNP could actively target inflammatory AS plaques depending on the neutrophil recruitment mechanism. RAW 264.7 macrophages were stimulated by LPS to induce an inflammation response, followed by incubation with DiI-labeled nanoparticles. As shown in Fig. 2G and H, intracellular fluorescence detected by flow cytometry showed that the internalization of both NP^DiI^ and NNP^DiI^ noticeably increased. However, NNP^DiI^-treated macrophages showed a 1.18- and 1.34-fold uptake of content than that of the NP^DiI^ group after 4 and 6 h, respectively. The CLSM results further proved the enhanced cellular internalization of NNP^DiI^, whereas the stronger DiI fluorescence remained within 6 h intracellularly (Fig. 2F). These results implied that biomimetic nanoparticles could mimic neutrophil function *in vivo* and can be targeted and accumulated in inflammatory areas of atherosclerosis.

Macrophage proliferation is observed in atherosclerosis development [45]. ST-treated local macrophage inhibition is an effective therapeutic strategy to prevent inflammation in atherosclerotic plaques. We investigated the cytotoxicity of neutrophil-mimicking NNP^ST^ in RAW 264.7 cells. In the absence of ST encapsulation, there was no considerable cytotoxicity after incubation with high concentrations of either NP or NNP (200 μg/mL, Fig. S4). Meanwhile, free ST, NP^ST^, and NNP^ST^ displayed a dose-dependent inhibitory effect on RAW 264.7 cells (Fig. 2B). Compared to free ST at higher concentration (i.e., 20 and 50 μg/mL), NNP^ST^ showed lower anti-cell proliferation ability owing to the slower and sustained ST release induced by neutrophil-membrane coating.

**Fig. 3:**
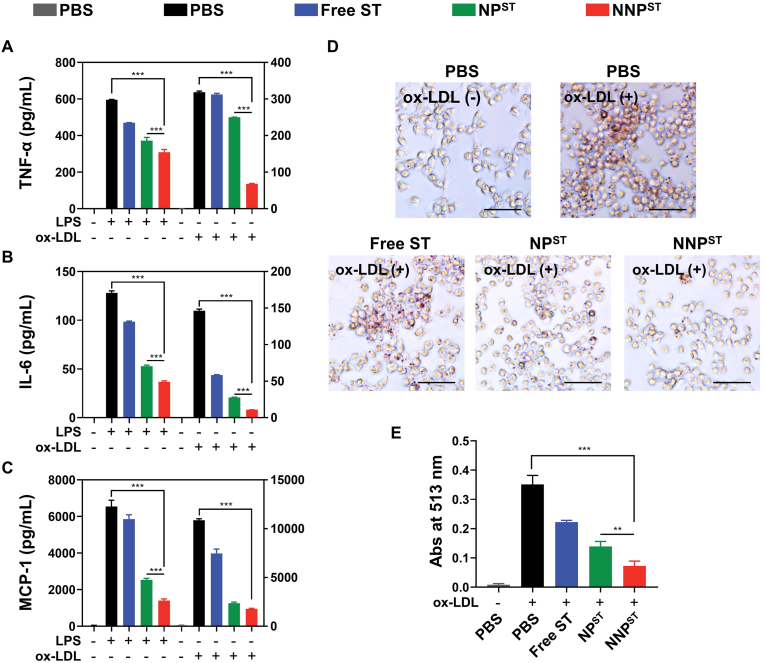
(A–C) ELISA assays for the typical inflammatory cytokines (i.e., TNF-α, IL-6 and MCP-1) secreted by LPS or ox-LDL treated RAW 264.7 cells (n = 3). (D) Optical microscopy images of ox-LDL induced foam cells formation with various treatments. The scale bar is 50 μm. (E) Quantitative content of extracted ORO from foam cells in each group (n = 3, mean ± SD). ∗∗*p* < 0.01 and ∗∗∗*p* < 0.001.

The inflammatory macrophages contribute to atherosclerosis progression by secreting large amounts of proinflammatory cytokines, and foam cells derived from ox-LDL-elicited macrophages are the hallmark of plaque formation in artery intima [6,7,25,32]. To confirm that the NNP^ST^ could alleviate inflammation, we induced RAW 264.7 cells using LPS or ox-LDL and measured the secreted contents of proinflammatory cytokines after differential treatments. As shown in Fig. 3A–C, free ST and ST-loaded nanoparticles reduced the secretion of TNF-α, IL-6, and MCP-1, and the lowest cytokines levels were observed in the NNP^ST^ treatment group. Additionally, NNP^ST^ decreased the number of macrophages showing immature dendritic-cell-like morphological changes, which were stimulated by either LPS or ox-LDL (Fig. S5). Further, we examined the attenuation of foam cell formation owing to NNP^ST^ using ORO staining. There were numerous ORO-positive cells in the ox-LDL-elicited group while the number of lipid droplets was reduced in free ST, NP^ST^, and NNP^ST^-treated macrophages (Fig. 3D and E). Compared with ST and NP^ST^-treated macrophages, NNP^ST^ substantially decreased the lipid-laden macrophages and attenuated immature dendritic-cell-like morphological changes, suggesting that NNP^ST^ reduced the uptake of ox-LDL by macrophages (Fig. S6). These results revealed that the antiatherogenic effect of NNP^ST^ could attenuate macrophage inflammatory responses and inhibit ox-LDL-induced foam cell formation.

### 3.3 *In vivo* homing to atherosclerotic plaque of NNP

**Fig. 4:**
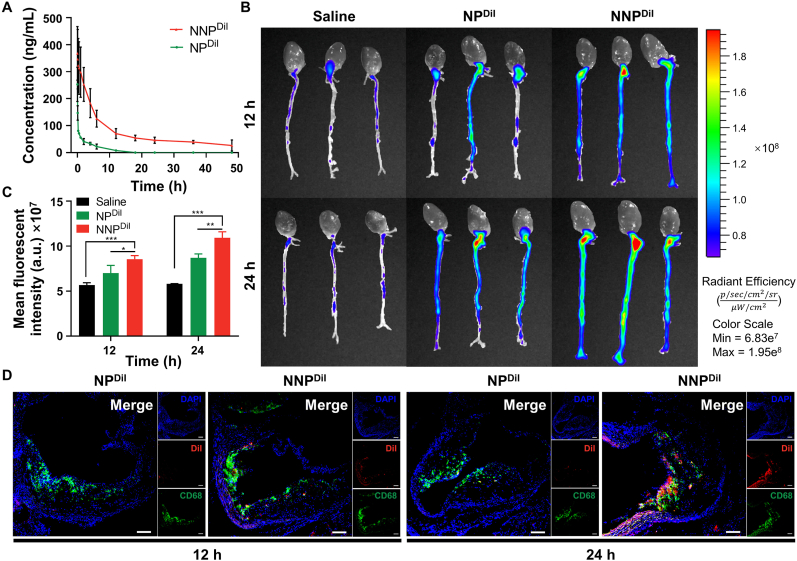
(A) *In vivo* blood circulation of NP^DiI^ and NNP^DiI^ within 48 h. *Ex vivo* fluorescence images (B) and quantitative DiI fluorescent signals (C) of the aorta at 12 and 24 h post-injection (n = 3). (D) Immunofluorescence analyses of aortic plaque areas (red: DiI; green: CD68^+^ macrophages; blue: nuclei). The scale bar is 100 μm ∗*p* < 0.05, ∗∗*p* < 0.01 and ∗∗∗*p* < 0.001. (For interpretation of the references to color in this figure legend, the reader is referred to the Web version of this article.)

Because the neutrophil-membrane-coated NNP could avoid ox-LDL uptake by macrophages, we assessed its long-term circulation in the bloodstream *in vivo*. The C57BL/6 mice were treated with a systemic injection of NP^DiI^ or NNP^DiI^, and their blood samples were collected from the orbital venous plexus at predetermined time points. The DiI concentration in the plasma was measured using a fluorescence spectrophotometer. As shown in Fig. 4A, the DiI concentration in the blood declined distinctly after the intravenous injection of NP^DiI^ from 252.61 ng/mL to 41.16 ng/mL after 2 h, which was ∼0.16-fold than that observed with NNP^DiI^ injection (252.86 ng/mL) after 2 h. Compared to NP^DiI^, NNP^DiI^, benefiting from the surface neutrophil membrane, achieved an evidently prolonged retention time in blood. At 24 and 48 h post-injection, the total DiI retention concentrations of NNP^DiI^ were 45.73 ng/mL and 26.52 ng/mL, respectively. Nevertheless, NP^DiI^ was rapidly cleared by the immune system *in vivo* owing to the absence of neutrophil membrane and there was only a trace fluorescence signal at 18 h post-injection.

Atherosclerosis is more likely to develop in the aortic sinus, arch and abdominal aorta areas because of abnormal blood flow patterns, which are frequently accompanied by inflammatory cell infiltration [3]. Considering the inflammation targeting of NNP *in vitro*, the ability of NNP^DiI^ to target atherosclerotic plaques was further validated *in vivo*. The ApoE^−/−^ mice were first fed a high-cholesterol diet for three months to induce AS. The DiI-labeled NP^DiI^ or NNP^DiI^ were administered *via* the tail vein at the DiI dose of 2.8 μg/mL. At predetermined time points, the dissected aorta showed varying fluorescence that increased over time, whereas no obvious fluorescence was found in the saline-treated group (Fig. 4B). Notably, the NNP^DiI^ group showed the most vigorous fluorescence after both 12 and 24 h post-injection. The mean fluorescent intensity in these regions (aortic sinus, arch and abdominal aorta areas) was 1.22- and 1.26-fold, respectively, than that found in NP^DiI^-treated cells (Fig. 4C), which was due to the amplified ELVIS effect [15]. Additionally, the fluorescence signal of both NP^DiI^ and NNP^DiI^ groups was primarily distributed in the liver following *i.v.* injection (Figs. S7A and B). Moreover, consistent with the *in vitro* findings, NNP^DiI^ are predominantly colocalized with inflammatory infiltrating CD68^+^ macrophages in aortic sinus, confirming the active targeting of atherosclerotic plaques *in vivo* (Fig. 4D).

**Fig. 5:**
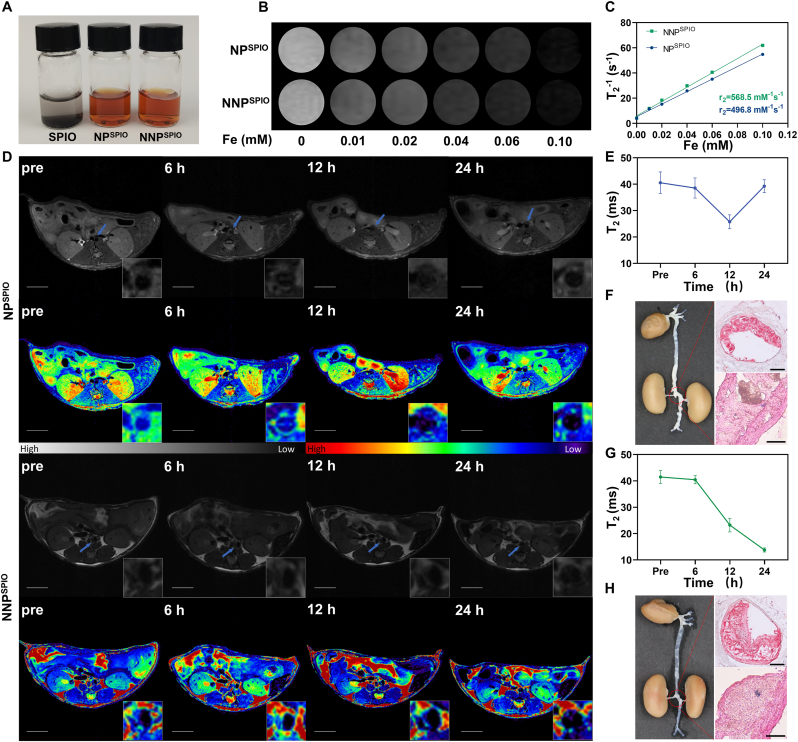
MRI performance of SPIO-loaded nanoparticles. (A) SPIO, NP^SPIO^ and NNP^SPIO^ suspension in ddH_2_O at room temperature. (B) T_2_-weighted images of NP^SPIO^ and NNP^SPIO^ at different Fe concentrations. (C) T_2_ relaxation rates of NP^SPIO^ and NNP^SPIO^. (D) Representative T_2_-weighted and the pseudo-color images in ApoE^−/−^ mouse at different time points before and post-injection with NP^SPIO^ or NNP^SPIO^. The scale bar is 5 mm. The mean T_2_ values of plaques after injection with NP^SPIO^ (E, n = 3) and NNP^SPIO^ (G, n = 3). Representative excised aorta and the renal artery branch sections stained with ORO and Prussian blue at 24 h post-injection with NP^SPIO^ (F) and NNP^SPIO^ (H). The scale bar is 200 μm. (For interpretation of the references to color in this figure legend, the reader is referred to the Web version of this article.)

Owing to superior soft tissue resolution, MRI could accurately detect and characterize atherosclerotic plaques. To visualize and monitor the targeting and enrichment of biomimetic nanoparticles within the plaque, oleylamine-modified SPIO was loaded into the inner PLGA core to improve its suspension in aqueous solution (Fig. 5A) The spin–spin relaxation time of NP^SPIO^ and NNP^SPIO^ was measured at various concentrations (Fig. 5B). The r_2_ of NP^SPIO^ and NNP^SPIO^ was 496.8 mM^−1^s^−1^ and 568.5 mM^−1^s^−1^ (Fig. 5C), respectively, indicating satisfactory negative contrast in T_2_-weighted MRI. Because of turbulent blood flow in the renal artery branch area of the abdominal aorta, atherosclerotic plaques are generally formed in the arterial wall. Accordingly, T_2_-Weighted and T_2_map sequences were acquired in this area to dynamically monitor SPIO-loaded nanoparticle deposition in the plaque. On T_2_-weighted MR images, atherosclerotic plaques exhibited local thickening of the arterial wall (Fig. 5D). In both groups, the mean T_2_ values of plaques showed no obvious changes before and 6 h post-injection. Although a slight reduction in the mean T_2_ value was detected in the NP^SPIO^ group at 12 h post-injection, it raised at 24 h post-injection again, probably because of the rapid clearance from blood circulation (Fig. 5G). Based on neutrophil-membrane modification induced anticirculatory clearance and preferential enrichment in atherosclerotic plaques, the NNP^SPIO^ group presented a time-dependent enhancement in T_2_ imaging. The local signal reduction of atherosclerotic plaque was evident at 12 h post-injection. At 24 h post-injection, the signal in most areas of atherosclerotic plaque declined considerably to be comparable to that of the blood flowing, and the mean T_2_ value of the plaque was reduced from 41.50 ms to 13.80 ms (Fig. 5E and G). Subsequently, we performed a histological analysis of aortic sections. ORO staining revealed large red-stained plaques in the renal artery branch area, which was consistent with the MRI results. Moreover, iron deposition (multiple blue-stained areas) was observed in the NNP^SPIO^ group after Prussian blue staining, implying NNP^SPIO^ enrichment in plaques (Fig. 5F and H, Figure S8). Neutrophil-membrane modification offers NNP to selectively deliver the imaging agent and therapeutic drug to inflammatory plaques for atherosclerosis theranostic.

**Fig. 6:**
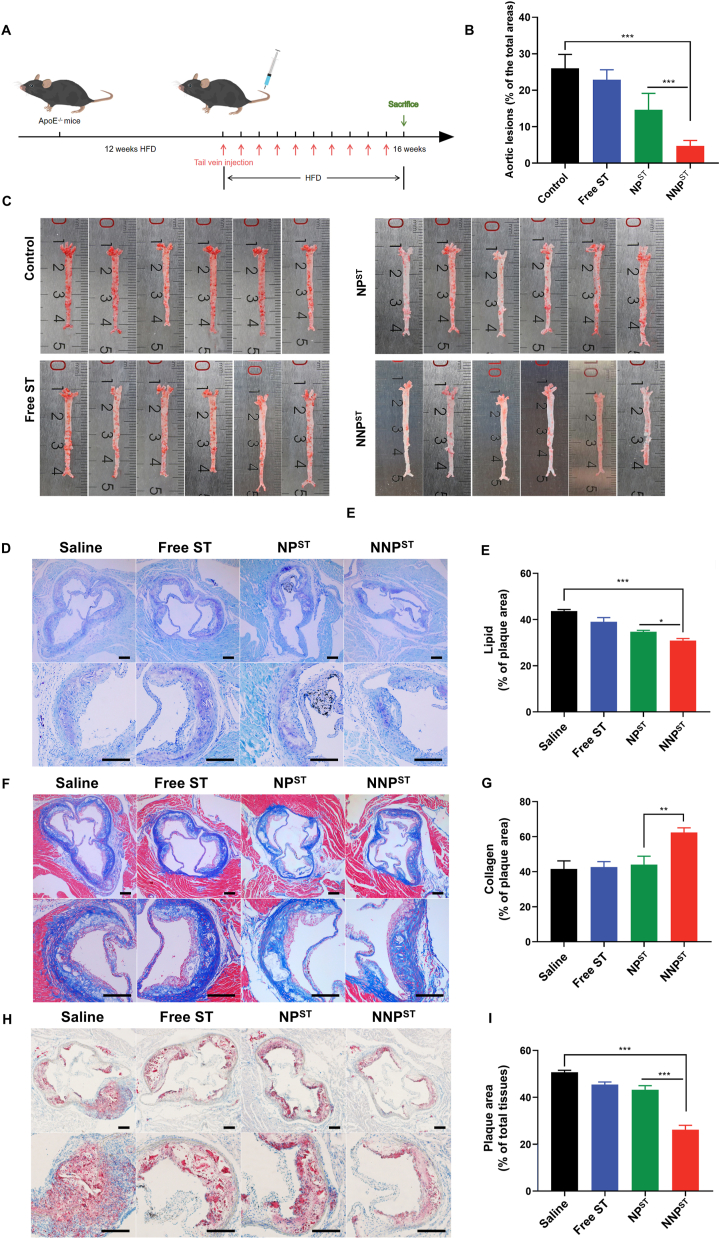
Therapeutic efficiency against AS in ApoE^−/−^ mice. (A) Schematic diagram of AS model development and treatment plan. (B) Quantitative analysis of the lesion areas collected from atherosclerotic mice (n = 6, mean ± SD, ∗∗∗*p* < 0.001). (C) The excised aorta tissues with ORO staining. Representative histological images and quantitative analysis of aorta root sections stained with ORO (D&E), toluidine blue (F&G), and Masson's trichrome (H&I) (n = 3, ∗*p* < 0.05, ∗∗*p* < 0.01, ∗∗∗*p* < 0.001). The scale bar is 200 μm. (For interpretation of the references to color in this figure legend, the reader is referred to the Web version of this article.)

Inspired by the payload accumulation in atherosclerotic lesions of neutrophil membrane–camouflaged nanoparticles, we investigated the therapeutic effects of NNP^ST^ against atherosclerotic development *in vivo*. After feeding a cholesterol-rich diet for three months, the ApoE^−/−^ mice were intravenously administered with saline, ST, NP^ST^, or NNP^ST^ ([ST] = 1 mg/kg) every 3 days (Fig. 6A). On day 30, the aortas were collected and stained with ORO to indicate plaque area by red region (Fig. 6B and C). Compared to the control group, the free ST and NP^ST^ group marginally improved the therapeutic effect. The ratio of plaque to the entire aortic area decreased from 26.02 % ± 3.81 %–22.86 % ± 2.78 % (free ST group) and 14.61 % ± 4.53 % (NP^ST^ group). Of note, there was a 21.27 % reduction in plaque in the NNP^ST^ treated mice, compared to the control group, suggesting an efficient inhibition of atherosclerosis progression. Moreover, we quantitatively evaluated atherosclerotic lesions by calculating plaque area/aortic area in slices (Fig. 6D and E) [46]. The lesion areas was a 24.57 % reduction after NNP^ST^ treatment, compared to the saline group.

In addition, we evaluated the therapeutic efficacy of NNP^ST^ using immunohistochemistry analyses to directly measure atherosclerotic plaque. Statins could improve atherosclerosis progression by reducing arterial lipid deposition and increasing collagen accumulation in plaques [9]. As displayed in Fig. 6F and G, plaques in the saline group protruded into the vascular lumen with notable lipid deposition, which accounted for 43.68 % ± 0.65 % of the plaques. Both free ST and NP^ST^ similarly reduced lipid deposition to 38.99 % ± 1.87 % and 34.71 % ± 0.61 %, respectively. However, in the neutrophil-mimicking NNP^ST^ group, a reduction in plaque area was observed, and the mean plaque area decreased to 30.89 % ± 0.88 % one month after treatment. Notably, an attenuated pathological plaque was most evident among different groups. Collagen accumulation in plaque could promote plaque stability and reduce clinical cardiovascular events [47]. Therefore, MASSON staining was used to detect the collagen content in plaque at the root of the aortic arch (Fig. 6H and I). The collagen content in the average plaque increased to 62.39 % ± 2.65 % after one month of NNP^ST^ treatment. Compared with the saline group, the collagen content in NP^ST^ and free ST groups increased to 44.06 % ± 4.78 % and 42.63 % ± 3.10 %, respectively, with no significant difference.

**Fig. 7:**
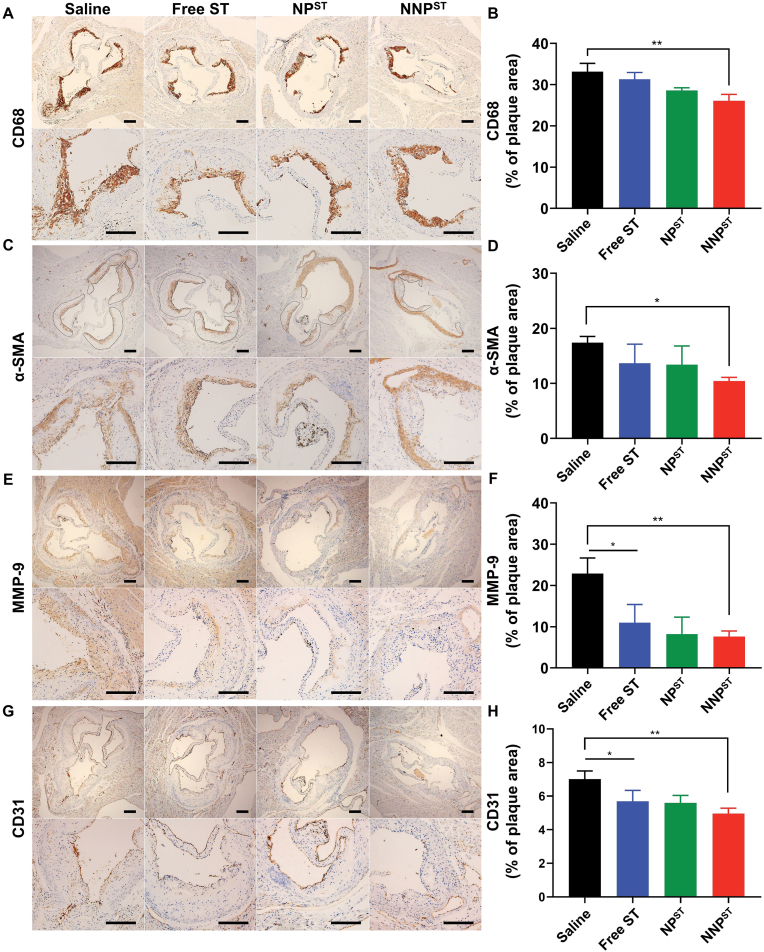
Representative immunohistochemistry staining images and quantitative analysis of aorta root sections of CD68 (A&B), α-SMA (C&D), MMP-9 (E&F) and CD-31 (G&H). ∗*p* < 0.05, ∗∗*p* < 0.01, ∗∗∗*p* < 0.001. The scale bar is 200 μm.

Subsequently, CD68 (macrophage markers) and α-SMA (SMCs markers) were employed for plaque labeling because the proliferation of both macrophages and SMCs positively correlated with plaque advancement [48,49]. As shown in Fig. 7A–D, the CD68 and α-SMA-positive cells in the NNP^ST^ group were lower than in the saline group. After the phagocytosis of lipids by macrophages, foam cells would produce excess MMP-9 [50]. There were extensive MMP-9-positive cells in the plaques of the saline, free ST, and NP^ST^ groups, indicating a fragile plaque phenotype and representing plaque stability (Fig. 7E and F). Compared to the angiogenesis of the saline group, CD31^+^ stained sections displayed decreased neovascularization in the plaques of the NNP^ST^ group (Fig. 7G and H). These results demonstrated that NNP^ST^ could substantially inhibit the development of atherosclerotic plaques increase the collagen content and reduce lipid deposition in plaques, providing a more powerful prognosis of plaque deterioration.

**Fig. 8:**
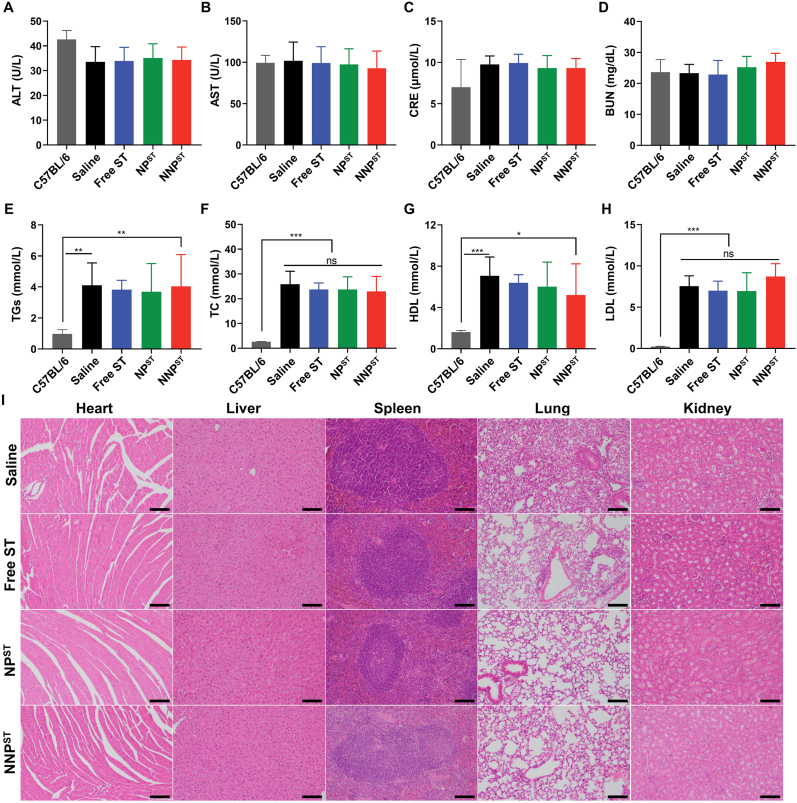
The blood concentration of ALT (A), AST (B), CRE (C), BUN (D), TGs (E), TC (F), HDL (G) and LDL (H) from the ApoE^−/−^ mice after the treatment (n = 6, mean ± SD). ∗*p* < 0.05, ∗∗*p* < 0.01, ∗∗∗*p* < 0.001 and *ns*, no significance. (I) Typical H&E staining images of major organs from ApoE^−/−^ mice after treatment. The scale bar is 100 μm.

To assess the biosafety of biomimetic NNP^ST^ on ApoE^−/−^ mice, their body weights were monitored during the treatment. Meanwhile, the pathological condition of major organs, routine blood analysis, blood biochemistry and blood lipid indexes were measured on day 30 after sacrificing the animal. All the groups exhibited no considerable differences in body weight and complete blood count (Figs. S9 and S10). Except for the creatinine level, which slightly increased, other liver and kidney function markers, such as alanine aminotransferase (ALT), aspartate aminotransferase (AST), and blood urea nitrogen (BUN), were normal and comparable following high-fat diet feeding and antiatherogenic therapy (Fig. 8A–D). Because of the high-fat diet, the high-density lipoprotein cholesterol (HDL), low-density lipoprotein cholesterol (LDL), total cholesterol (TC), and triglycerides (TGs) associated with atherosclerotic progression were higher than those of wild-type C57BL/6 mice (Fig. 8E–H), which is in agreement with previous reports [51,52]. Moreover, H&E staining in the NNP^ST^ group showed no appreciable tissue damage in major organs (Fig. 8I). These results verify that our biomimetic NNP^ST^ might function as a theranostics platform with good biocompatibility for AS treatment.

## 4 Conclusion

We have successfully constructed a biomimetic nanosystem derived from neutrophil membranes and explored accurate atherosclerosis theranostics. NNP exhibited many advantages. First, the innate targeting and migration capabilities of neutrophil membranes helped nanoparticles evade the phagocytic system and actively target inflammatory areas, prolonging ST accumulation and retention in atherosclerotic lesions. In addition, the PLGA inner core remarkably improves the water solubility of ST and realizes sustained release. Second, NNP^SPIO^ could function as a highly efficient MRI contrast agent for dynamically visualizing atherosclerosis plaques. Third, the released ST exhibited enhanced anti-inflammatory efficiency to delay atherosclerosis progression. Finally, pathological examination and clinical serum biochemistry analysis confirmed that the biomimetic nanocomposite showed good biosafety *in vivo*. Such a neutrophil membrane–coating strategy provides new avenues to fabricate biomimetic nanomedicines for treating atherosclerosis and other inflammatory diseases.

## CRediT authorship contribution statement

**Ningnannan Zhang:** Writing – review & editing, Writing – original draft, Visualization, Data curation. **Tianzhu Zhang:** Writing – review & editing, Writing – original draft, Methodology. **Jintang Feng:** Writing – original draft, Visualization. **Jian Shang:** Supervision. **Beibei Zhang:** Software, Investigation. **Qingyang Dong:** Project administration. **Zhang Zhang:** Project administration, Conceptualization. **Chunyang Sun:** Resources, Project administration, Conceptualization.

## Declaration of competing interest

The authors declare the following financial interests/personal relationships which may be considered as potential competing interests:Zhang Zhang reports financial support was provided by 10.13039/501100001809National Natural Science Foundation of China. Ningnannan Zhang reports financial support was provided by 10.13039/501100001809National Natural Science Foundation of China. Zhang Zhang reports financial support was provided by 10.13039/501100006606Natural Science Foundation of Tianjin. Zhang Zhang reports financial support was provided by Health science and Technology project of Tianjin. Zhang Zhang reports financial support was provided by Tianjin Key Medical Discipline (Specialty) Construction Project. Zhang Zhang reports financial support was provided by Wu Jieping Medical Foundation-special Fund for Clinical Research. Chunyang Sun reports financial support was provided by 10.13039/501100001809National Natural Science Foundation of China. Chunyang Sun reports financial support was provided by Excellent Young Scientist Foundation of 10.13039/501100010104Tianjin Medical University General Hospital. If there are other authors, they declare that they have no known competing financial interests or personal relationships that could have appeared to influence the work reported in this paper.

## Data Availability

The authors do not have permission to share data.
