# Supplementary material for: Application of therapeutical nanoparticles with neutrophil membrane camouflaging for inflammatory plaques targeting against atherosclerosis
Source: Mater Today Bio. 2024 Dec 10;30:101397. doi: 10.1016/j.mtbio.2024.101397 (PMC11722182; doi:10.1016/j.mtbio.2024.101397)
Supplement: Multimedia component 1 [file mmc1.docx]

**Application of Therapeutical Nanoparticles with Neutrophil Membrane Camouflaging for Inflammatory Plaques Targeting against Atherosclerosis**

Ningnannan Zhang^†, #^, Tianzhu Zhang^†, #^, Jintang Feng^†, #^, Jian Shang^†^, Beibei Zhang*^¶^*

, Qingyang Dong^‡, *^, Zhang Zhang^†, *^, Chunyang Sun^†, *^

^†^ Department of Radiology and Tianjin Key Laboratory of Functional Imaging, Tianjin Medical University General Hospital, Tianjin 300052, P.R. China

^‡^ Department of Environmental Medicine, Tianjin Institute of Environmental and Operational Medicine, Tianjin 300050, P.R. China

*^¶^* Department of Magnetic Resonance Imaging, The First Affiliated Hospital of Zhengzhou University, Zhengzhou 450002, P.R. China

*^#^* These authors contributed equally to this work.

E-mail: dongqy_Meta@126.com (Q. Dong); filea1249@sina.com (Z. Zhang); chysun@tmu.edu.cn (C. Sun)

**Methods**

**Characterization of nanoparticles**

The size and zeta potential of NMV, NP^ST^, NP^SPIO^, NNP^ST^ and NNP^SPIO^ were determined using a Nano-ZS90 dynamic light scattering (DLS, Malvern, UK) with a He-Ne laser (λ = 633 nm) at a scattering angle of 90° at 25 °C. 50 μL of nanoparticles solution at a concentration of 500 µg/mL were dropped onto a copper mesh, and stained with 2% phosphotungstic acid. Subsequently, the morphology of various nanoparticles was observed using a transmission electron microscope at 80 kV (TEM, HT7700, Hitachi, Japan).

**Characterization of membrane makers proteins**

Coomassie blue staining and Western blotting assay were used to verify the proteins on the particles. Briefly, the NP^ST,^ NP^SPIO^, prehomogenated neutrophils, extracted neutrophil membrane, NNP^ST^ and NNP^SPIO^ were firstly lysed in radio immunoprecipitation assay (RIPA) lysis buffer and centrifuged at 20,000 g for 5 min at 4 ºC. The supernatant was collected and subjected to the BCA protein assay kit. Next, the proteins were mixed in sodium dodecyl sulfate (SDS) loading buffer and heated at 95 ºC for 15 min. These samples were run on 10% SDS-polyacrylamide gel at 80 V for 0.5 h and then at 120 V for 1 h. The resulting polyacrylamide gel was stained with Coomassie blue overnight and imaged.

For western blot analysis, equivalent amounts of protein from each sample were separated by SDS-PAGE and then transferred the proteins onto the polyvinylidene difluoride membrane, which was blocked at rt for 1 h. Subsequently, the blots were incubated with the following antibodies against PSGL-1, CD31, TLR4 and LFA-1 for 12 h at 4 ºC, respectively, and then incubated with the horseradish peroxidase (HRP)-conjugated secondary antibodies. The protein signals were observed under a molecular imager (ChemiDoc, Bio-Rad, USA).

***In vitro* cytotoxicity assay**

*In vitro* cytotoxicity of various nanoparticles against RAW 264.7 cells was evaluated by CCK-8 assays. RAW 264.7 cells were seeded in 96-well plates at a density of 1 × 10^4^ cells per well. After incubation for 12 h, the cells were treated with different concentrations of NP or NNP for 24 h. Then, the cell media were removed and replaced with 100 μL CCK-8 solution. After 1 h, the absorbance at 450 nm was measured by a BioTek microplate reader.

**Proliferation Inhibition of macrophages *in vitro***

RAW 264.7 cells were used to detect *in vitro* antiproliferation of NP^ST^ and NNP^ST^. Briefly, the cells were inoculated in 96-well plates (1 × 10^4^ cells per well) and cultured for 24 h, then the cells were further incubated with fresh medium containing ST, NP^ST^ or NNP^ST^ at different concentrations. After incubation for 24 h, the cell viability was measured by the CCK-8 assay.

***In vitro* anti-inflammatory effects**

RAW 264.7 cells were cultured in 24-well plates at a density of 1×10^5^ cells and treated with ST, NPST or NNPST ([ST]= 5 μg/mL) for 12 h, respectively. Subsequently, PBS, LPS (100 ng/mL) or ox-LDL (100 μg/mL) were added and incubated with macrophages for 24 h, respectively. The tumor necrosis factor-α (TNF-α), monocyte chemoattractant protein-1 (MCP-1) and interleukin-6 (IL-6) in the supernatants was detected by ELISA kits. The cell morphology was examined by the optical microscope.

**Foam cell formation inhibition**

RAW 264.7 cells were treated with 100 ng/mL of LPS for 24 h and then incubated with ST, NP^ST^ or NNP^ST^ ([ST]= 5 μg/mL) for 12 h, followed by co-incubation with 100 μg/mL of ox-LDL for 48 h. After washing with PBS for three times, the macrophages were fixed with 4% paraformaldehyde and stained with ORO (0.3%). The intracellular ORO was quantified using a UV-Vis spectrometer after extraction with isopropanol (60%).

**Colocalization study**

For fluorescence imaging experiments, the DiO (green) labeled neutrophil membrane was then coated on the surface of NP^DiI^. For the colocalization study, RAW 264.7 cells were cultured in DMEM medium containing 10% FBS at 37 °C with 5% CO_2_. 200 μg of NNP^DiI^ were added to RAW 264.7 cells. After incubation for 4 h, the cells were washed with PBS for three times, followed by staining with DAPI. The colocalization images were captured by confocal laser scanning microscopy (CLSM, LSM-800, Zeiss, Germany).

**Statistical analysis**

All data are expressed as mean ± SD in this study. Student's t-test was performed for comparison between two groups, and one-way analysis of variance (ANOVA) analysis was conducted for comparing multiple groups followed by a Tukey post hoc analysis. In all data, a P-value of＜0.05 was considered statistically significant (**p* < 0.05, ***p* < 0.01, ****p* < 0.001).


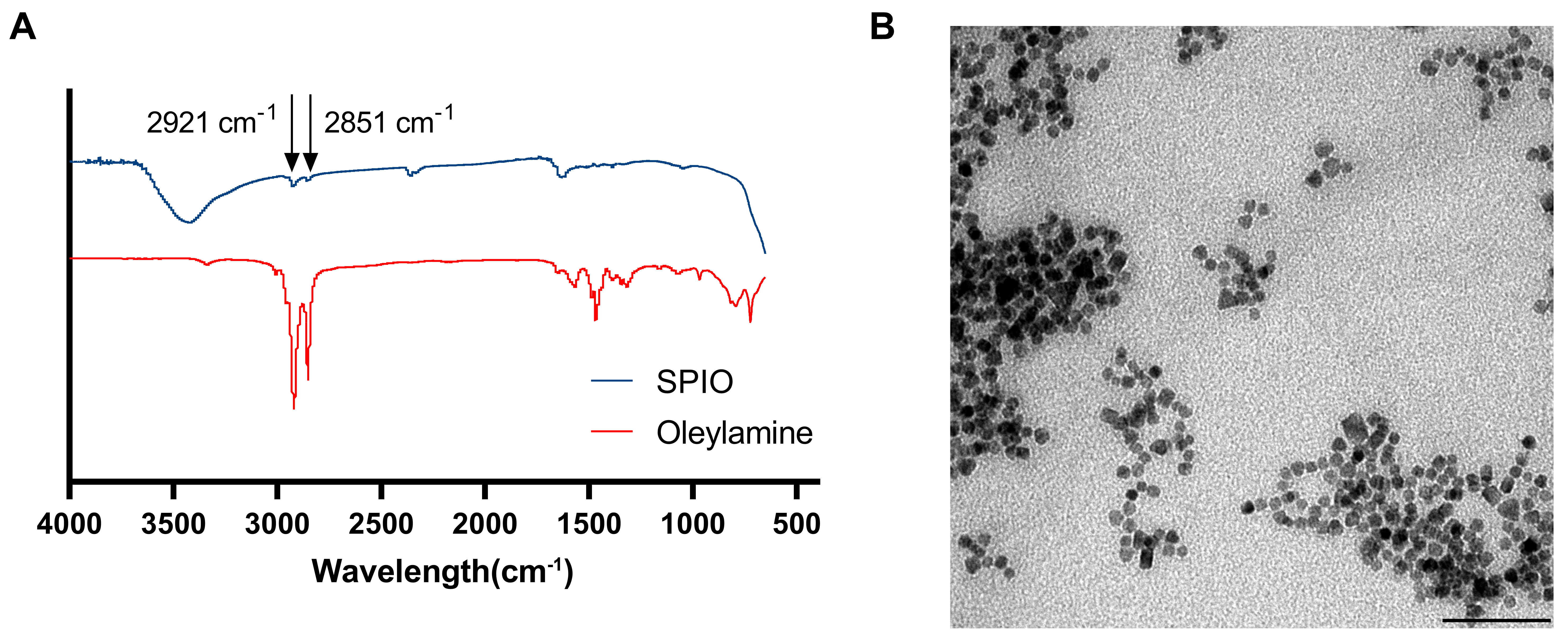


**Figure S1.** FT-IR spectra (A) and TEM (B) of SPIO nanoparticles. The scale bar is 50 nm.


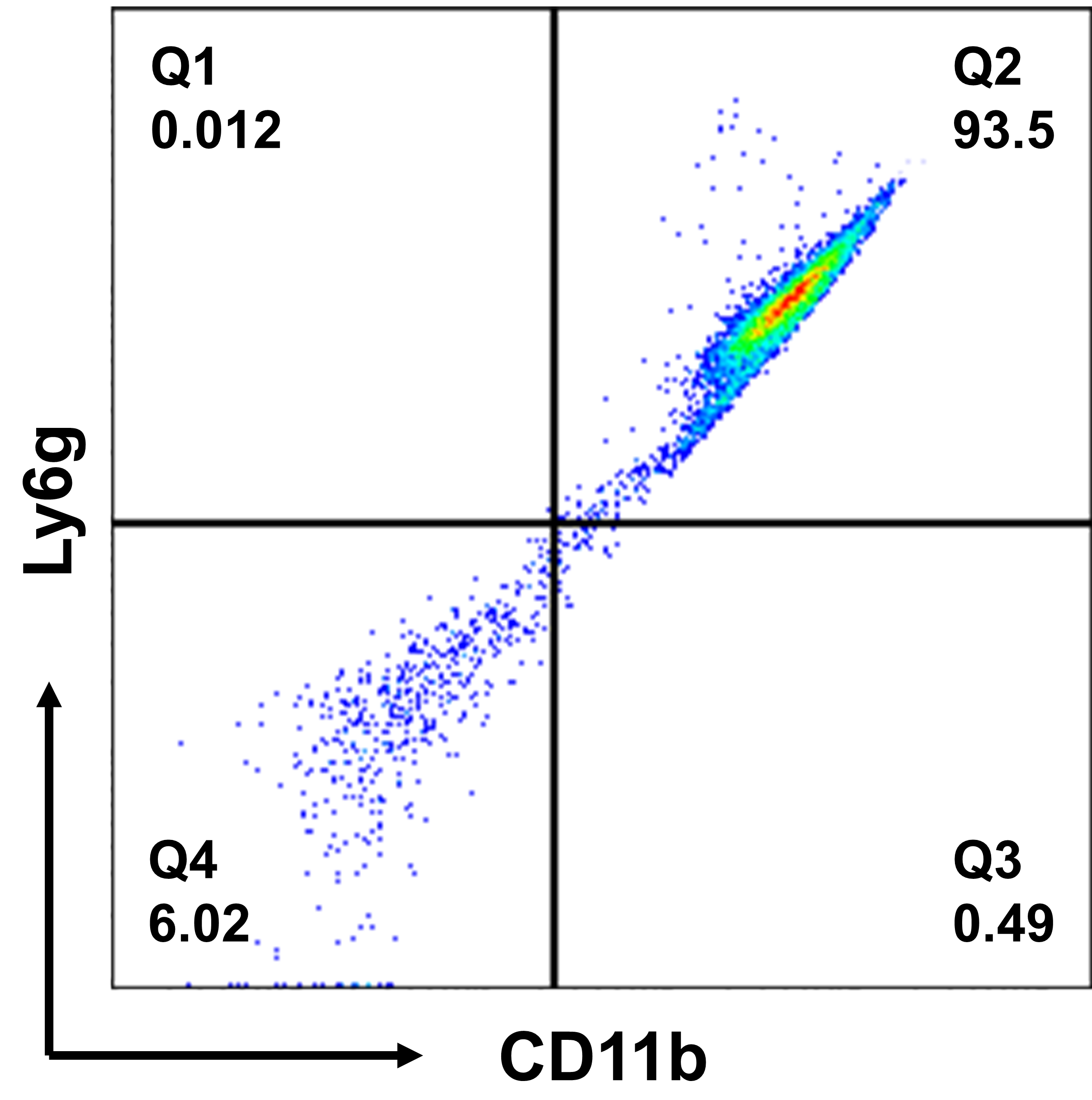


**Figure S2.** The neutrophils purity in the cells extracted from bone marrow of mice after isolation.


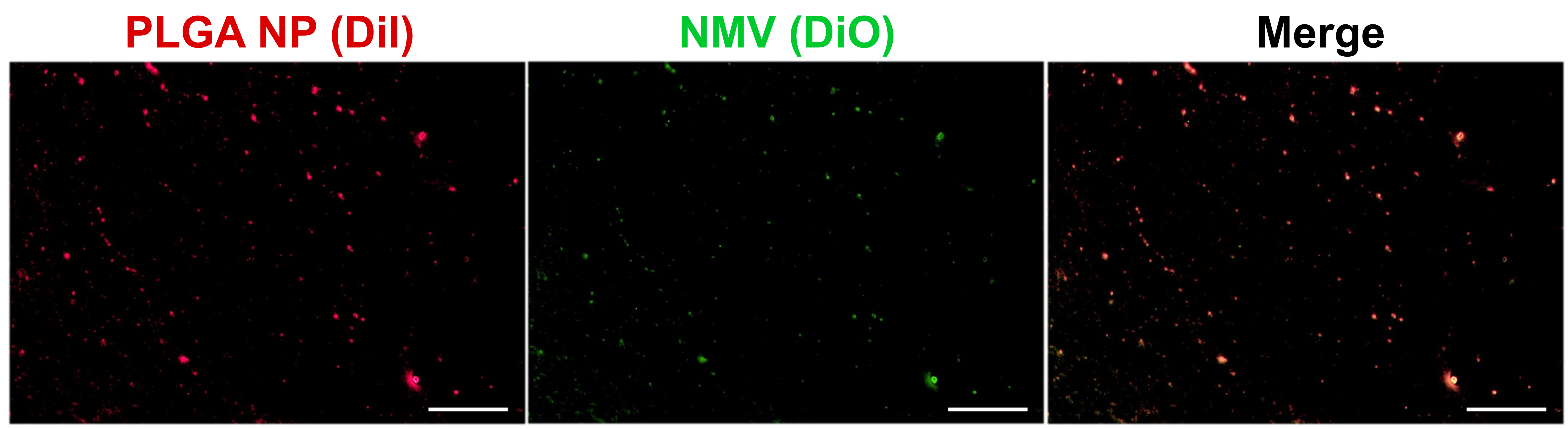


**Figure S3.** CLSM images of DiI labeled PLGA NP (red), DiO labeled NMV (green) and merged NNP^DiI^ (yellow). The scale bar is 10 μm.


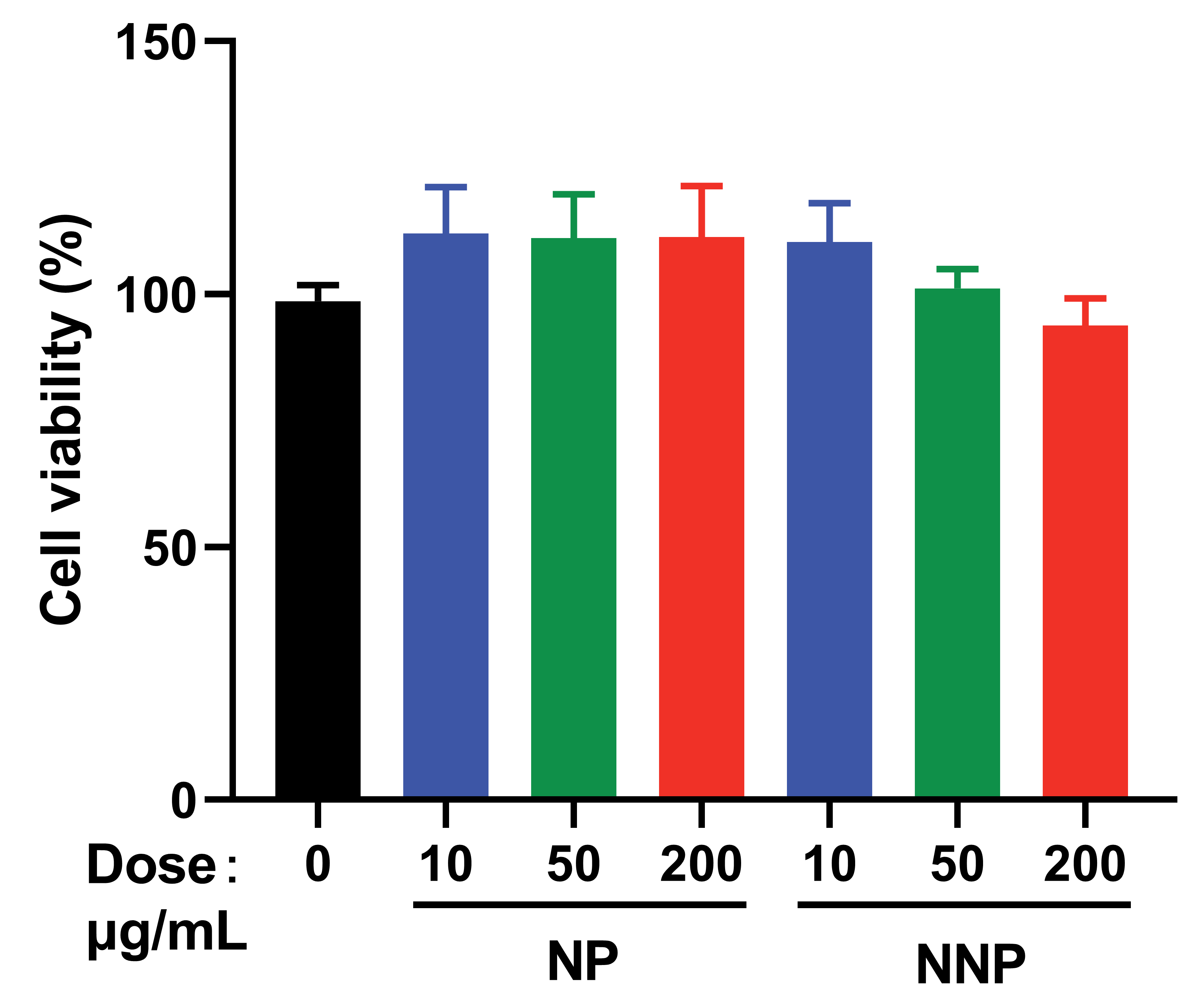


**Figure S4.** The viabilities of RAW 264.7 cells after the treatment with NP or NNP at different concentrations for 24 h (n = 3).


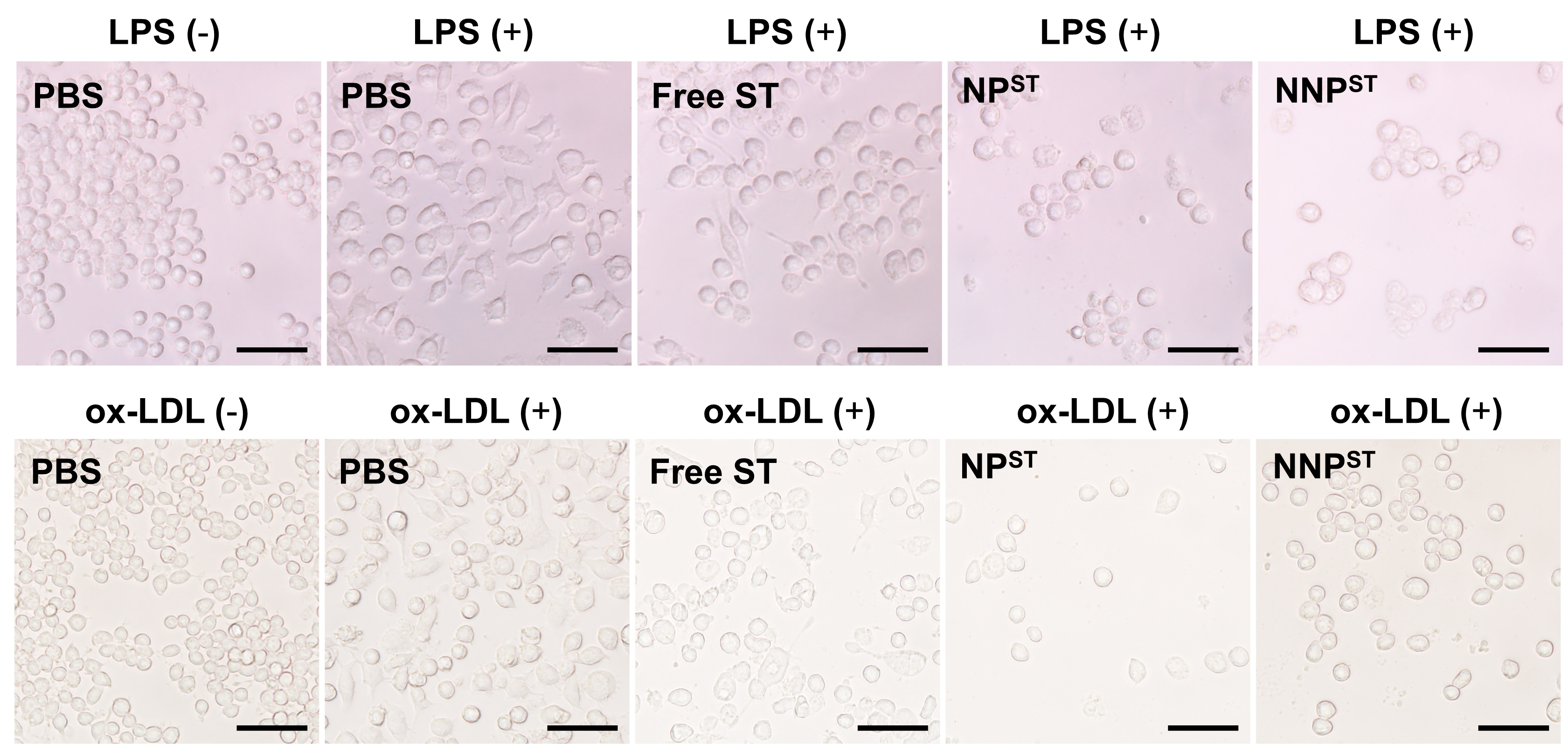


**Figure S5.** The phenotype changes of RAW 264.7 cells after the treatment with various formulations for 24 h ([ST]= 5 μg/mL). The scale bar is 50 μm.


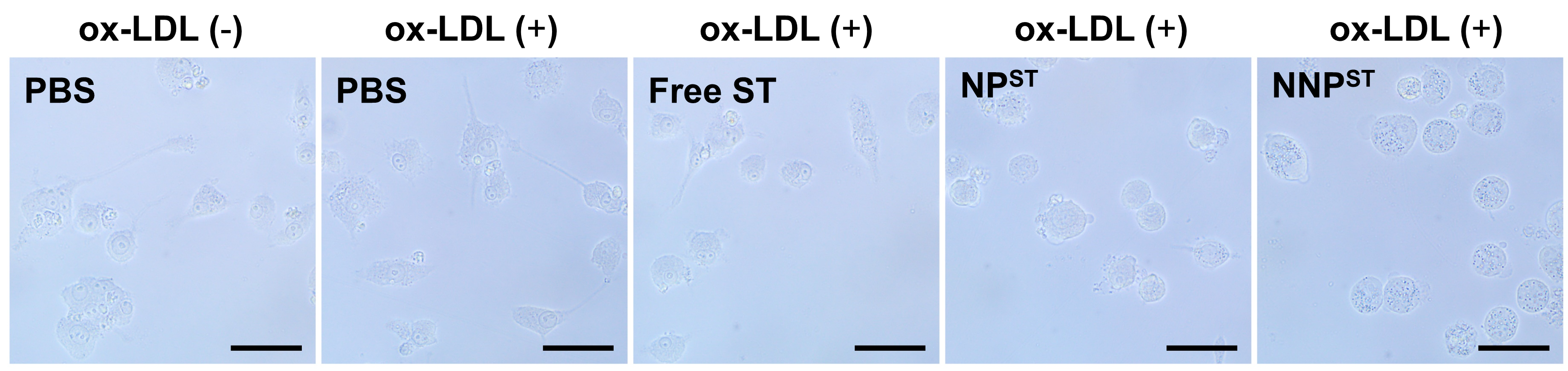


**Figure S6.** The phenotype changes of RAW 264.7 cells after the treatment with various formulations for 48 h ([ST]= 5 μg/mL). The scale bar is 50 μm.


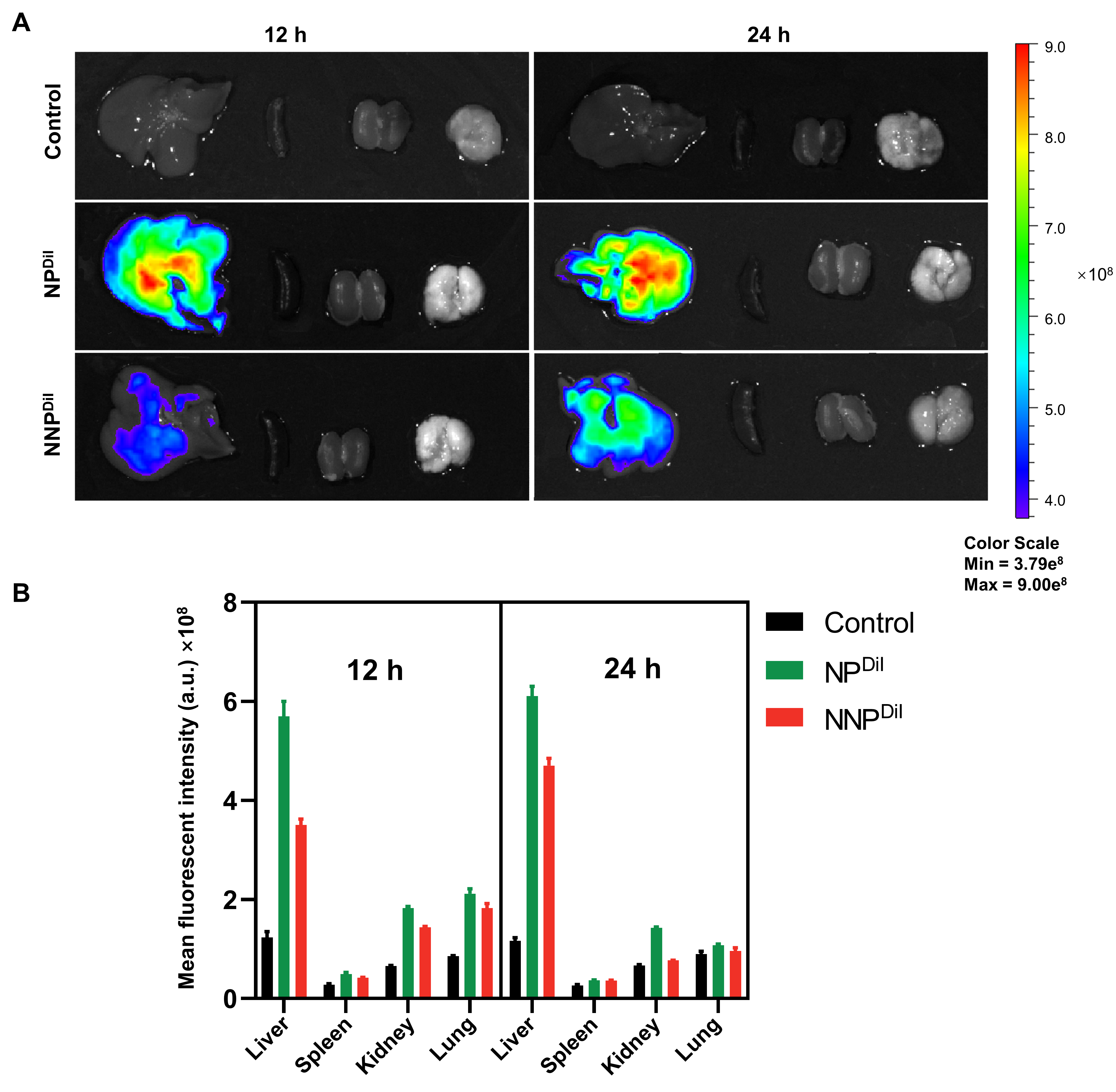


**Figure S7.** *Ex vivo* fluorescence images (A) and DiI fluorescent signal (B) in different organs at 12 and 24 h post-injection (n = 3, mean ± SD).


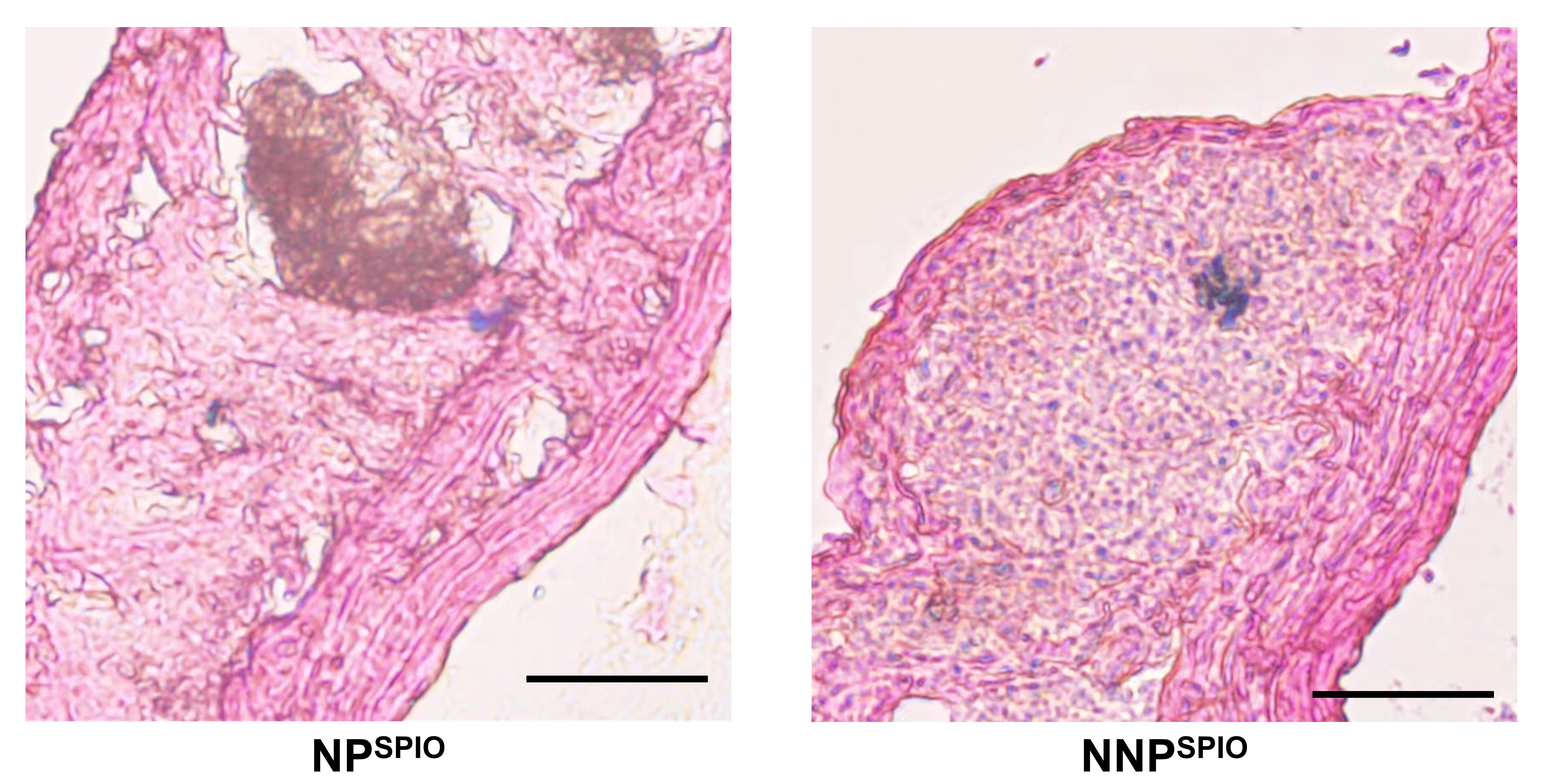


**Figure S8.** Representative excised aorta and the renal artery branch sections stained with Prussian blue at 24 h post-injection with NP^SPIO^ and NNP^SPIO^. The scale bar is 200 μm.


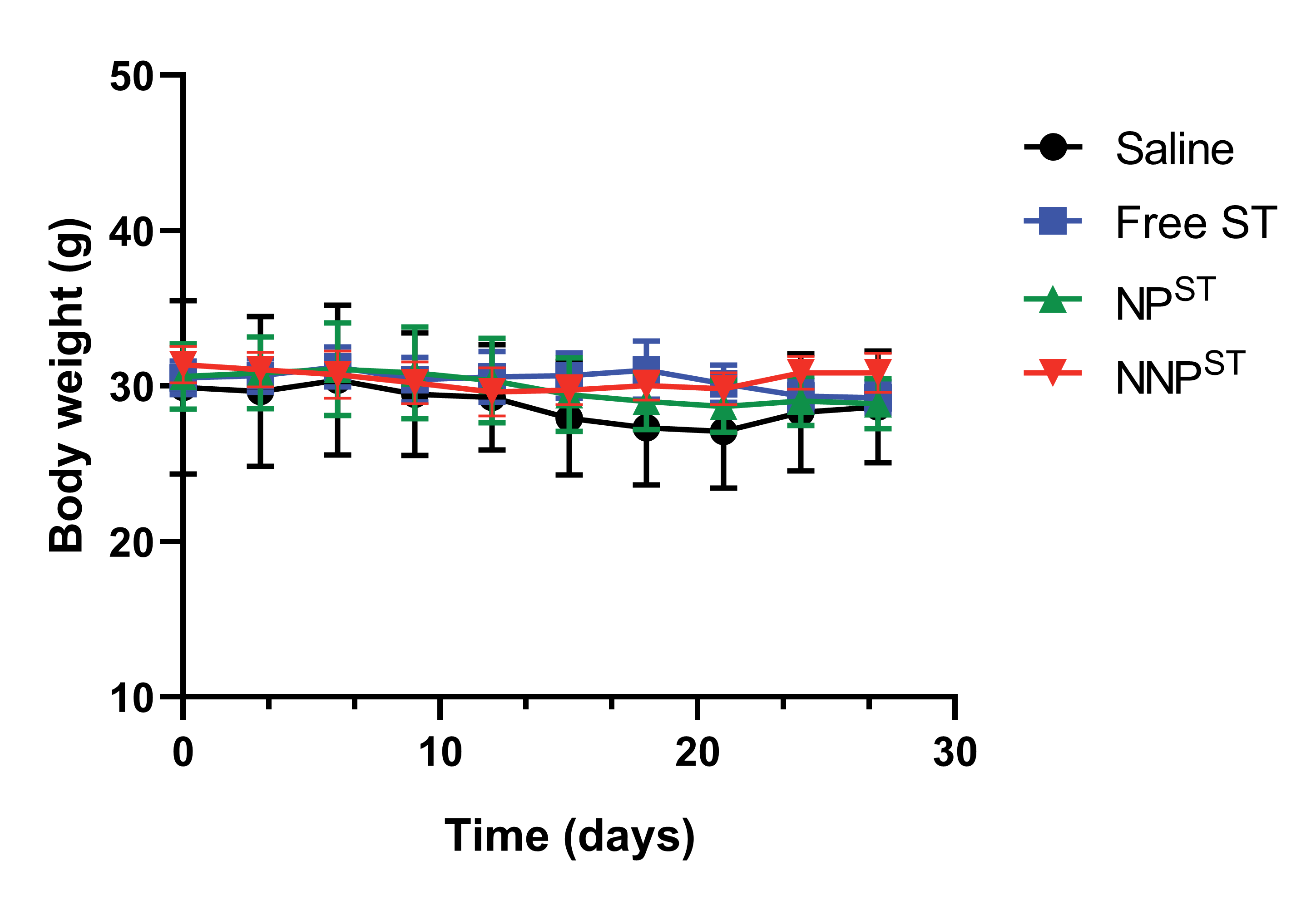


**Figure S9.** The body weight of atherosclerotic mice treated with various formulations over time (n=6).


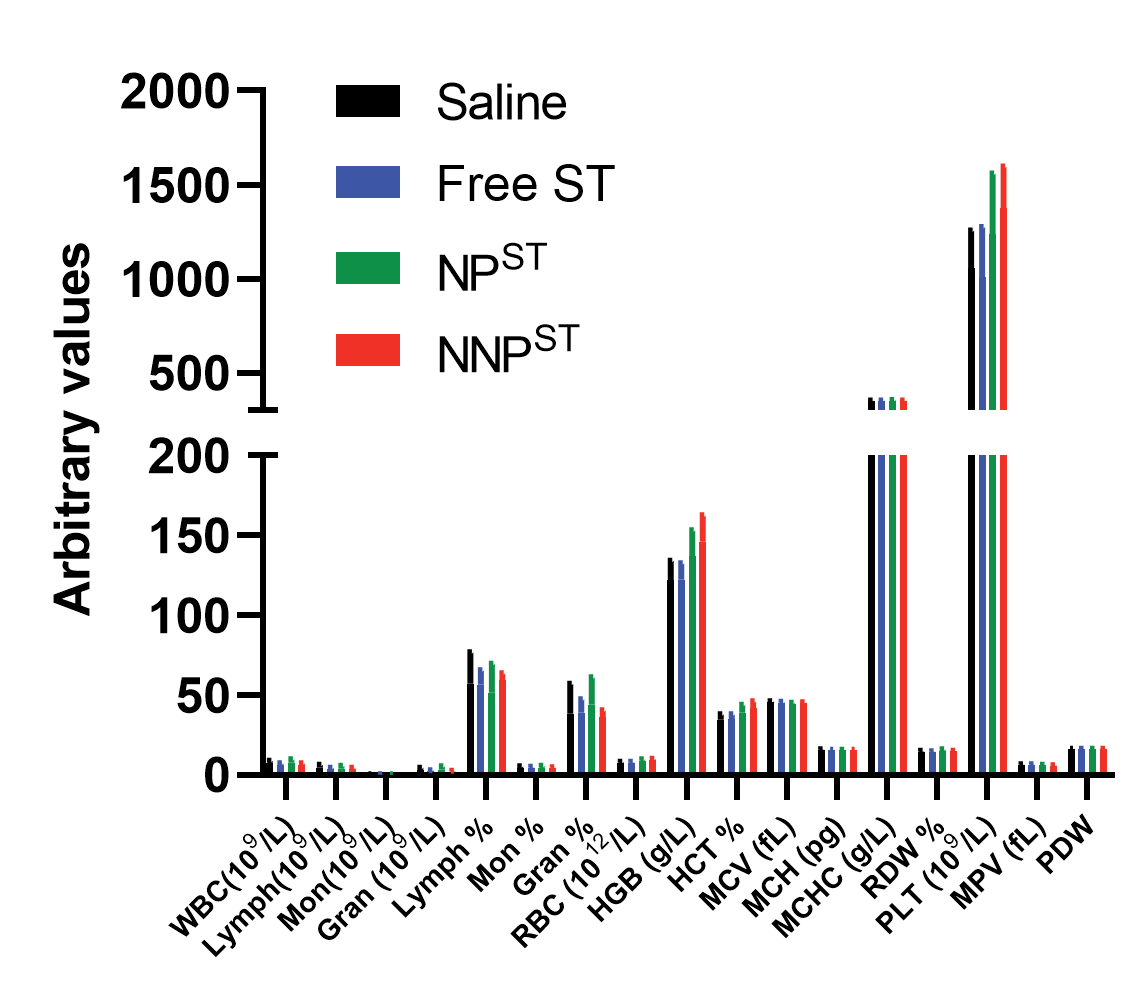


**Figure S10.** Complete blood count in atherosclerotic mice after one-month treatment (n = 6).
